# Decoding Voluntary Movement of Single Hand Based on Analysis of Brain Connectivity by Using EEG Signals

**DOI:** 10.3389/fnhum.2018.00381

**Published:** 2018-11-05

**Authors:** Ting Li, Tao Xue, Baozeng Wang, Jinhua Zhang

**Affiliations:** ^1^Shaanxi Key Laboratory of Clothing Intelligence, School of Computer Science, Xi'an Polytechnic University, Xi'an, China; ^2^State and Local Joint Engineering Research Center for Advanced Networking and Intelligent Information Services, School of Computer Science, Xi'an Polytechnic University, Xi'an, China; ^3^State Key Laboratory for Manufacturing Systems Engineering, School of Mechanical Engineering, Xi'an Jiaotong University, Xi'an, China

**Keywords:** voluntary movement decoding, EEG, brain connectivity, brain functional network, hierarchical linear model

## Abstract

Research about decoding neurophysiological signals mainly aims to elucidate the details of human motion control from the perspective of neural activity. We performed brain connectivity analysis with EEG to propose a brain functional network (BFN) and used a feature extraction algorithm for decoding the voluntary hand movement of a subject. By analyzing the characteristic parameters obtained from the BFN, we extracted the most important electrode nodes and frequencies for identifying the direction of movement of a hand. The results demonstrated that the most sensitive EEG components were for frequencies delta, theta, and gamma1 from electrodes F4, F8, C3, Cz, C4, CP4, T3, and T4. Finally, we proposed a model for decoding voluntary movement of the right hand by using a hierarchical linear model (HLM). Through a voluntary hand movement experiment in a spiral trajectory, the Poisson coefficient between the measurement trajectory and the decoding trajectory was used as a test standard to compare the HLM with the traditional multiple linear regression model. It was found that the decoding model based on the HLM obtained superior results. This paper contributes a feature extraction method based on brain connectivity analysis that can mine more comprehensive feature information related to a specific mental state of a subject. The decoding model based on the HLM possesses a strong structure for data manipulation that facilitates precise decoding.

## Introduction

Research about decoding the neurophysiological signals from the human brain aims to translate them into control signals for external devices. In the ideal state of a Brain-Computer Interface system (BCI), it can accurately discern a subject's body movement intents, and output smooth, accurate control to external devices, such as neural prosthesis. Motor control is the systematic regulation of movement in organisms that possess a nervous system. This process requires cooperative interaction between the central nervous system and the musculoskeletal system.

Brain signals have been adopted in BCI, including electrocorticography (ECoG) (Miller et al., 2010; Pistohl et al., 2012), electroencephalography (EEG) (Wolpaw and McFarland, 2004; Bradberry et al., 2010), functional magnetic resonance imaging (fMRI) (Yoo et al., 2004; Sitaram et al., 2007), magnetoencephalography (MEG) (Boostani and Moradi, 2003; Bradberry et al., 2009), and near-infrared spectroscopy (NIRS) (Coyle et al., 2007) signals. Each of these signals has its own strengths and limitations. The selection of one over another for brain imaging applications will depend on the cost of the equipment as well as the spatial and temporal resolution required (Min et al., 2010). Currently, ECoG signals with high quality and spatial resolution constitute the fundamental way to realize high communication rates in BCI (Wilson et al., 2006). Numerous studies have been performed using ECoG to extract control signals for BCI (Lal et al., 2005; Schalk et al., 2007). Researchers (Hochberg et al., 2012) have successfully demonstrated the direct control of robotic prosthetic limbs with many degrees of freedom using ECoG signals from the motor cortex of patients with tetraplegia. However, ECoG is limited owing to its invasiveness and requires clinical surgery to place electrodes on the surface of the human brain. For noninvasive approaches, EEG offers good temporal resolution but poor spatial resolution, while NIRS provides only moderate temporal resolution and also moderately better spatial resolution (Nicolas-Alonso and Gomez-Gil, 2012). Indeed, recent studies reported successful application of NIRS-BCI for facilitating the communication of patients in a completely locked-in state (Chaudhary et al., 2017). Until now, a number of hybrid BCI studies have demonstrated the effectiveness of the combinatory use of different modalities or paradigms. Researchers have also demonstrated the feasibility of MEG and FMRI (Breitwieser et al., 2010). Current technology for recording MEG and fMRI is both expensive and bulky, making it unlikely for practical applications in the near term. fNIR is potentially cheaper and more compact. However, both fMRI and fNIR are based on changes in the cerebral blood flow, an inherently slow response (Khan et al., 2014).

EEG records signals generated by the neuroelectrical activities on the scalp and its noninvasiveness makes it more practically usable than ECoG in BCI. As the analysis methods become more efficient and precise for the noninvasive mode, researchers and engineers gradually realize that the noninvasive mode will be the paradigm with more acceptance in BCI applications. This is one of the most important motivations for the development of the study. However, a key problem related to noninvasive BCI technologies is the limited number of control modes obtained from decoding the movements of body parts (for example, upper and lower limbs) (Liao et al., 2014). It remains unclear whether noninvasive EEG signals have sufficient information to decode the kinematics parameters of voluntary movements (Liao et al., 2014). Noninvasive EEG-based BCI has been developed to decode a user's movement intention based on the markers of active brain involvement in the preparation of the desired movement. However, it is generally concluded that the signal-to-noise ratio, band width, and information content of neural data acquired via noninvasive EEG are insufficient to extract detailed information about natural, multijoint movements of the upper limbs (Bradberry et al., 2009). To enable EEG decoding to achieve the same effect as ECoG decoding, improving motion feature extraction and decoding model design is essential.

Current state-of-the-art BCIs have employed two types of EEG to detect motor intention, i.e., movement-related cortical potentials (MRCPs) and sensory motor rhythms (SMRs). Because of the low number of orders and delay in the order of seconds, SMR-based BCIs still lack natural and intuitive control (Müller-Putz et al., 2016). MRCPs are slow EEG fluctuations-associated. Movement intention detection through MRCPs has been shown to have relatively short latencies. For upper limb movements, MRCP was analyzed for discriminating movement directions and trajectories (Bradberry et al., 2010; Müller-Putz et al., 2016; Pereira et al., 2017), and grasp types (Jochumsen et al., 2016). More recently, some BCIs have combined MRCPs and SMRs to boost their decoding performance (Lew et al., 2012; Ibáñez et al., 2014; Úbeda et al., 2017).

The use of a linear regression model to fit EEG and velocity profiles requires that these two temporal signals remain in the same frequency range. In addition, in the method of linear regression, researchers use a correlation to evaluate the fitting of the reconstructed trajectory and the measured trajectory. This evaluation method could lead to overly optimistic decoding results. Moreover, the nonlinear nature of correlation makes EEG signals at low frequencies more appropriate for decoding. However, there is no definitive proof that EEG signals at high frequencies do not contain information about dexterous movements. EEG signals are also nonlinear and non-Gaussian. The mathematical relation between EEG and voluntary limb movements would be complex and largely dependent on the properties of EEG features used for decoding. An increasing number of theoretical and empirical studies approach the function of the human brain from a network perspective (Sporns et al., 2005). The motor areas of the cerebral cortex involved in motor execution consist of the primary motor cortex (M1) and several premotor areas, including the supplementary motor area (SMA), presupplementary motor area (pre-SMA), and ventral and dorsal parts of the premotor cortex (PMC). The prefrontal and frontal cortices play a significant role in cognitive and motor events that instantiate action planning and programming (Decety, 1995). Researchers in this field are studying the neural mechanism of limb movement control to find methods for decoding complex movements by using the whole EEG information (Babiloni et al., 2017; Yu et al., 2017) and correlation characteristics at different levels (Filho, Attux and Castellano, 2018), for example, graph metrics (Cavallo et al., 2016). Synchronization between different brain regions is known to be an essential feature of cognitive processing in general. Different cognitive tasks are associated with different connectivity patterns between brain regions (He'tu et al., 2013). In noninvasive BCI research, several measures of connectivity have been developed for analyzing EEG recordings (Nair et al., 2003; Lacourse et al., 2004). These studies focus on the differences in the connectivity patterns in motor execution. These connectivity patterns should be detectable from EEG recordings, and thus offer a new type of feature space for inferring a subject's intention. For movement decoding and BCI control, it is not effective to only consider frequency bands and small subsets of electrodes known to be relevant to motor execution. It is important to properly address possible volume conduction effects, not confine the analysis to a small subset of electrodes, and consider a broad range of frequency bands. Based on this consideration, feature extraction algorithms with the ability of Macro data processing is required for BCIs.

Based on the discussion above, we propose a method for decoding voluntary hand movement based on an analysis of brain connectivity using EEG signals. We carried out an experiment about the single direction movement of a human hand in 3D space, and synchronously recorded the EEG signal and kinematic data of the hand. By analyzing the characteristic parameters obtained from the BFN, we extracted the most important electrode nodes and frequencies for identifying the direction of hand movement. The EEG data and the parameters of the BFN, both of which are synchronized with hand movement, possess a nested structure. We formed a model of voluntary hand movement decoding based on a hierarchical linear model (HLM). Finally, we performed a voluntary hand movement experiment in the spiral trajectory. The Poisson coefficient between the measurement trajectory and the decoding trajectory was used as a test standard to compare with the traditional multiple linear regression model (Bradberry et al., 2010). The main content of the current paper can be divided into four parts. In section Experiment, the experiments are described in detail. The process of brain connectivity analysis and hierarchical linear regression decoding are explained in section Calculation. In section Results, the important results are presented. The main contribution of the present paper is to verify the effectiveness of feature extraction based on functional brain connectivity analysis and propose an effective decoding model based on the HLM. The core novelties of the current study are as follows.

- This study focused on decoding voluntary hand movement, but with certain restricted modes of motion, e.g., center-outreaching task and limb movement according to cue.
- Using brain connectivity analysis as the feature extraction method, we identified the frequencies and electrodes with significant effects for recognizing the moving direction of the hand.
- To consider as many elements for decoding as possible, we used a hierarchical linear model (HLM) to elucidate the mathematical relation between the EEG signals and the kinematic parameters of the hand.

## Materials and methods

This study was carried out in accordance with the recommendations of Xi'an Jiaotong University Approval for Research Involving Animals, Comments of the laboratory animal care committee, Xi'an Jiaotong University. The protocol was approved by the Comments of the laboratory animal care committee, Xi'an Jiaotong University. All subjects gave written informed consent in accordance with the Declaration of Helsinki.

### Data collection

**Figure 1:**
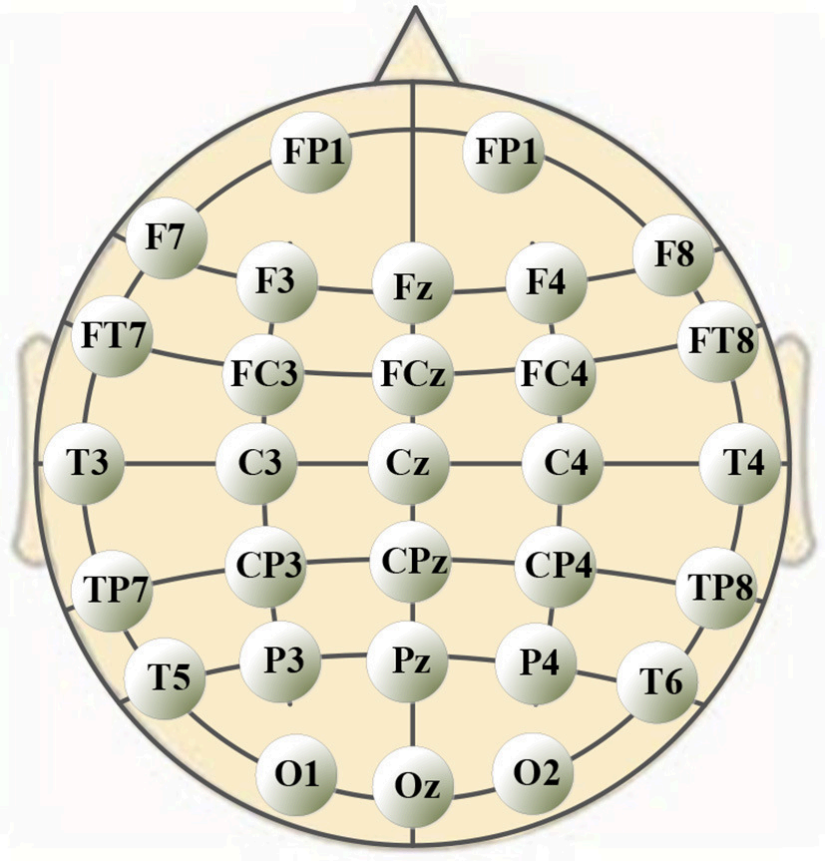
Positions of 30-channel EEG electrodes on subject's scalp.

A 40-channel NuAmps system (NeuroScan, Inc. Sterling, USA) was used for data recording at a sampling rate of 1,000 Hz. Electrode impedances were kept below 5 kOhm for all electrodes. EEG signals were recorded from 32 electrodes (shown in Figure 1), with the ground electrode at Fz. The reference electrode A1 was on the left ear. Four additional electrodes were used to record horizontal and vertical EOGs. Scan 4.5 performed online EOG artifact rejection. A 50 Hz notch filter suppressed line noise.

We used the PST IRIS optical tracking/measurement system (PS-Tech, Amsterdam, Netherlands) to measure and record the kinematics parameters of voluntary hand movement. Table 1 represents the performance of the PST IRIS system used in the experiment. After the PST IRIS software entered the working state, one participant stood inside the tracking range of PST IRIS, and swung the right arm according to the instruction.

**Table 1:** The performance of PST IRIS in the experiment.

**Accuracy**	**Position: < 0.5 mm RMSE:Orientation: < 1 deg RMSE2**
Latency	15–25 ms (depending on the shutter time and the filter settings)
Sampling rate	120 Hz, adjustable to 30, 60, 120 Hz
Tracking distance	50 cm−5 m, up to 7 m
Tracking DOF	6 degrees of freedom in all movement space

*RMSE, root-mean-square error*.

### Experiment

Ten participants (3 female, 7 male) without previous experience in EEG experiments participated in the study. All subjects were right-handed, with a mean age of 26.3 years (variance was 3.3). Before the formal experiment, all subjects took the preliminary training on upper arm movement. In this training, a subject learned how to guide herself/himself in a comfortable standing position and keep the other parts of body in a state of inexertion when the right arm waved voluntarily.

**Figure 2:**
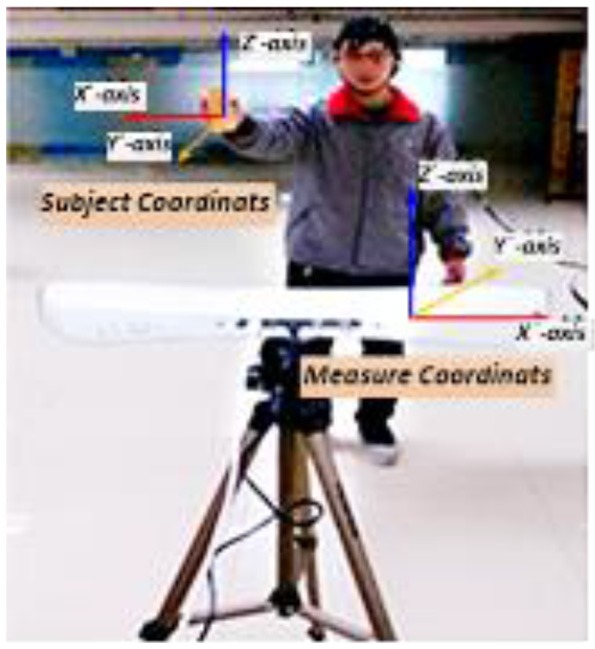
Synchronous recording experiment of hand motion trajectory and EEG signals. (1) Subject's right hand moved right or left; the PST IRIS system recorded positive or negative trajectory coordinates on the X-axis. (2) Subject's right hand moved close to or away from body; the system recorded negative or positive trajectory coordinates on the Y-axis. (3) Subject's right hand moved up or down; the system recorded positive or negative trajectory coordinates on the Z-axis.

The experiment was performed in two stages. Data collected in these two stages were used in feature extraction from the BFN and decoding model training/testing respectively. As shown in Figure 2, the “measure coordinate” was defined by the PST IRIS system, which recorded the kinematic trajectory of a subject's right hand during right arm winging. In the process of data collection, subjects tried to avoid body movements other than that of the right arm for reducing myoelectric interference. To ensure precise synchronization between EEG signals and hand movement data, we used the absolute timestamp to complete the synchronization.

#### Experiment 1: voluntary hand movement along single coordinate axis

**Figure 3:**
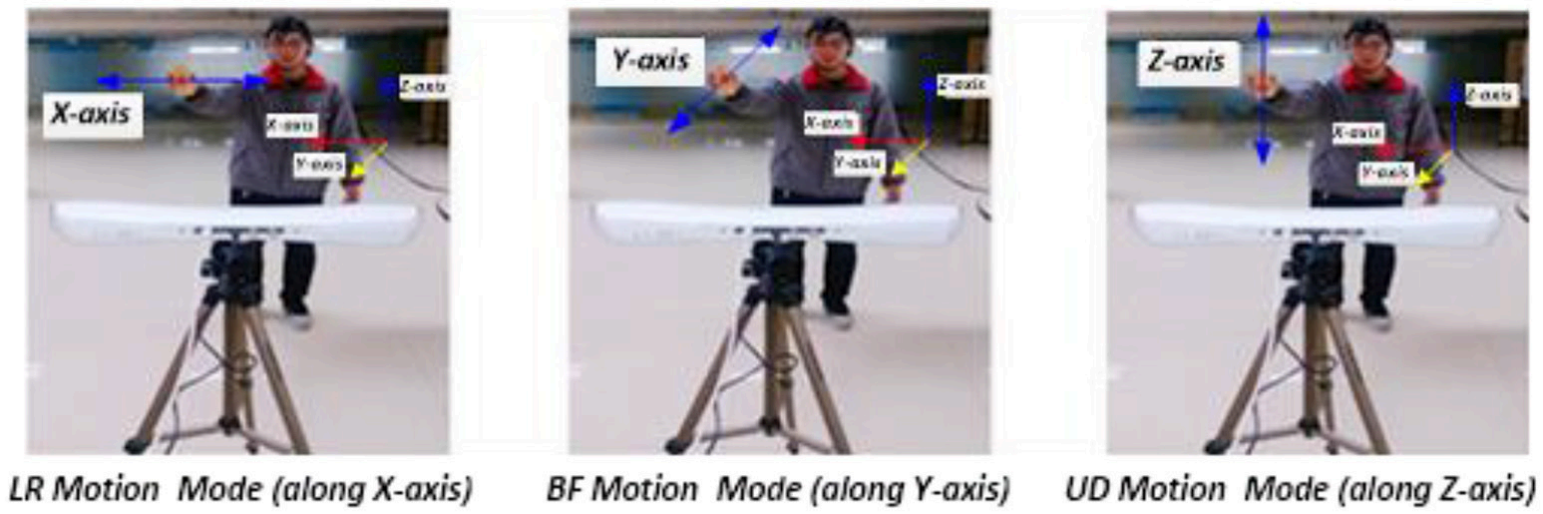
Three motion modes in Experiment 1.

Three single-direction hand motion modes were defined according to the axis direction: UD (up and down) movement on the Z-axis, LR (left and right) movement on the X-axis, and BF (backward and forward) movement on the Y-axis (Figure 3).

In this stage, a subject kept one degree of freedom of the shoulder joint. The elbow and wrist joints were not moved as much as possible. The acceleration of the hand changed mainly in a single direction. A subject's hand moving to the left (or up, or backward) and back to the original point was considered one unit. The test of each movement contained 5 units. Each motion mode was tested 20 times. In this process, subjects took a break anytime (1–2 s), when they felt tired. Excluding the rest time, the mean duration of each movement was 201.3 s (100 units). The subject was allowed to rest for 2–5 min between the different motion mode tests. The start and end points of the movement, the distance, and the speed were all determined by the subject. In total, each subject was tested 60 times (This experiment only recorded the motion data of the right hand. No test has been designed for the left hand for now).

#### Experiment 2: voluntary hand movement in spiral trajectory

**Figure 4:**
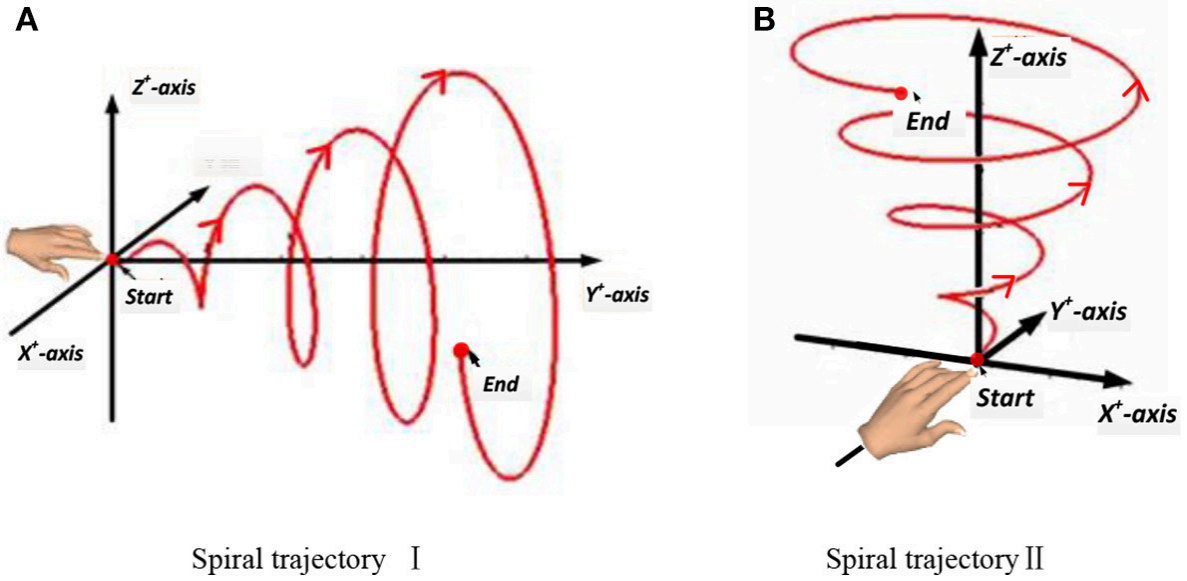
Two spiral trajectories in Experiment 2. **(A)** Spiral trajectory I. **(B)** Spiral trajectory II.

In this experiment, a subject made a spiral motion in front of the body with the right hand. In Figure 4, according to the “measure coordinate,” the first spiral movement was made along the Y axis, drawing a spiral from the near side of the body to the far side. The second spiral movement was made along the Z-axis, drawing an upward spiral from below. The start and end points of the movement, the distance, and the speed were all determined by the subjects, but the hand's spiral trajectory was required to be as round and smooth as possible.

In this stage, the subject kept three degrees of freedom of the shoulder joint, two degrees of freedom of the elbow joint, and no degree of freedom of the wrist joint. The acceleration of the hand changed in the three directions of the axes. The subjects were required to complete all movements while standing, and except during the spiral movements of the hand, the body was as still as possible. Each spiral movement was repeated 40 times. The mean duration of each spiral movement was 5.91 s. Excluding the rest time, the mean duration of each spiral movement was 238.4 s.

### Calculation

The full computation included two parts: parameter validation and trajectory decoding model training. Parameter validation consisted of four steps: Morlet wavelet decomposition, wavelet coefficient correlation calculation, BFN construction, and analysis of network characteristic parameters. Trajectory decoding model training was performed in two steps: EEG characteristic component extraction, and trajectory decoding model training and testing.

#### Preprocessing

The preprocessing flow of the EEG with Neuroscan mainly included the removal of DC components, visual inspection of data, deletion of channels and data segments with serious disturbances, and 50 Hz notch filtering. The postprocessing flow of the EEG with EEGLAB includes baseline correction, EOG and EMG artifact removal, and band-pass filtering (0–55 Hz).

#### Brain functional network

In this study, the Morlet continuous wavelet transform was used to decompose the EEG signals. According to the characteristic EEG frequency bands, the wavelet coefficients were extracted from each electrode and stored separately as a wavelet coefficient matrix. The eight characteristic frequencies are delta (1–3 Hz), theta (4–7 Hz), alpha1 (8–9 Hz), alpha2 (10–12 Hz), beta1 (13–17 Hz), beta2 (18–30 Hz), gamma1 (31–40 Hz), and gamma2 (41–50 Hz). We used Spearman's rank correlation coefficient as the scale to evaluate the spectral correlation between each pair of EEG electrodes. For certain characteristic frequency bands, the correlation matrix P channel was defined as follows:

(1) $$\begin{array}{l} \;\begin{array}{cccc} \;C_{1} & \;C_{2} & \;\cdots & \;C_{N} \end{array}\\ P_{channel}=\begin{array}{c} C_{1}\\ C_{1}\\ \vdots \\ C_{N} \end{array}\left[\begin{array}{cccc} 0 & p_{1,2} & \cdots & p_{1,N}\\ p_{2,1} & 0 & & p_{2,N}\\ \vdots & & \ddots & \vdots \\ p_{N,1} & p_{N,2} & \cdots & 0 \end{array}\right], \end{array}$$

where Cn, *n* = {1, 2 …, N}, represents the electrodes, with each node corresponding to the electrode position during EEG signal acquisition. P _i_, _j_, i,j ∈ {1,…,N}, represents the correlation coefficient of the wavelet coefficients from electrode nodes i and j. To generate a BFN with the small-world characteristic, the correlation coefficient matrix must be filtered by a threshold. In this study, we used the method of network cost to determine the threshold. Network cost *C*_*G*_ is an abstract concept that represents the cost of measuring and constructing the network. The equation for network cost *C*_*G*_ is given by

(2) $$C_{G}=\frac{K}{N\left(N-1\right)/2}$$

where *N* and *K* are the number of nodes and links. Here, the brain functional network (BFN) is actually an undirected weighted graph, and the weights between the electrode nodes are determined by using the following equation:

(3) $$a_{ij}=\left(p_{ij}-C_{G}\right)\;for\;i\neq j,$$

where *a*_*i*_, _*j*_ is the weight between electrode nodes *i* and *j*. When *a*_*i*_, _*j*_ < 0, the weight is set as 0. A threshold-processed correlation coefficient matrix is used to construct the EEG BFN. The BFN *F*_*net*_ is defined by the weight matrix as follows:

(4) $$\begin{array}{l} \;\begin{array}{cccc} C_{1} & \;C_{2} & \;\cdots & C_{N} \end{array}\\ F_{net}=C\left[\begin{array}{cccc} 0 & a_{1,2} & \cdots & a_{1,N}\\ a_{2,1} & 0 & & a_{2,N}\\ \vdots & & \ddots & \vdots \\ a_{N,1} & a_{N,2} & \cdots & 0 \end{array}\right], \end{array}$$

The weight matrix of the BFN is a symmetric matrix. The weight on the diagonal represents the weight of each node to itself, and a value of 1 would result in network loopback. Therefore, the weight of each node to itself is set to 0. Network characteristic parameters, namely, average correlation coefficient, average node degree, average path length, and clustering coefficient are calculated. The average correlation coefficient is the average of all weights. According to the definition in Equation (4), the average correlation coefficient P is calculated as follows:

(5) $$P=\frac{\sum a_{i,j}}{2n}\;i=1,\cdots ,N;j=1,\cdots ,N.$$

Since this BFN is an undirected network, the weight between any two nodes is the same regardless of the direction. When calculating the other three network coefficients, the nonzero correlation coefficients between the nodes are set to 1, indicating an existing path between the nodes—that is, the original weight *a*_*i*_, _*j*_ > 0, and *a*_*i*_, _*j*_ is set to 1. Otherwise, *a*_*i*_, _*j*_ is set to 0. After the network weight is converted to binary, the average node degree and the average correlation coefficient are calculated by using Equation (5). The shortest distance between any two nodes dist(i,j) is defined as the path length between these two nodes. The average of all node pairs' path lengths is defined as the average path length calculated as follows:

(6) $${\text{dist}}_{\text{c}}=\frac{2}{N(N-1)}\sum_{i\leq N}\sum_{j>i}dist\left(i,j\right).$$

If a node has k edges, then the maximum number of edges between the *k* nodes connected by those *k* edges is $\frac{k\left(k-1\right)}{2}$. The clustering coefficient C(p) of one node is its number of real edges divided by its maximum number of edges. The average of all the node clustering coefficients is the network clustering coefficient calculated as follows:

(7) $$\text{C}\left(\text{p}\right)\text{=}\frac{3\left(\text{K}-2\right)}{4\left(\text{K}-1\right)}{\left(1-\text{P}\right)}^{3},$$

where *K* is the number of adjacent points that may generate connections to a node, and *P* is the probability of reconnection of edges. Reconnection refers to an endpoint of an edge in the network remaining unchanged, while the other end is randomly selected as one node in the network.

#### Hierarchical linear regression decoding

We adjusted the EEG data and BFN parameters into a nested structure based on actual data relationships. Based on the hierarchical linear regression, we designed a voluntary movement decoding model using the EEG data and BFN parameters as inputs.

Level 1 of the voluntary hand movement decoding model is described by the following equations.

(8) $$x\left(t\right)=V_{x}+\sum_{n\;=\;1}^{N}\sum_{s\;=\;0}^{L}U_{snx}S_{n}\left(t-s\right)+\omega_{x}$$

(9) $$y\left(t\right)=V_{y}+\sum_{n\;=\;1}^{N}\sum_{s\;=\;0}^{L}U_{sny}S_{n}\left(t-s\right)+\omega_{y}$$

(10) $$z\left(t\right)=V_{z}+\sum_{n\;=\;1}^{N}\sum_{s\;=\;0}^{L}U_{snz}S_{n}\left(t-s\right)+\omega_{z}$$

Here, *x*(*t*), *y*(*t*), and *z*(*t*) are the coordinates of the hand at time point *t*. *N* is the number of characteristic electrodes extracted from BFN. *L* is the number of sampling points. *S*_*n*_(*t*−*s*) are the EEG data collected from electrode *n* at time point *t*−*s*. *U*_*snx*_, *U*_*sny*_, and *U*_*snz*_ are the slopes of the regression equation for the corresponding coordinates of electrode *n* at time point *t*−*s*. *Vx, Vy*, and *Vz* are the intercepts of the coordinates' regression equations. *ω*_*x*_, *ω*_*y*_, and *ω*_*z*_ are residuals.

Level 2 of the voluntary hand movement decoding model is described as follows.

(11) $$V_{x}=a_{vx}+\sum_{k=0}^{L}\left(c_{vkx}C_{\tau -k}+p_{vkx}P_{\tau -k}\right)+e_{vx}$$

(12) $$U_{snx}=a_{ux}+\sum_{\phi \in \Phi }\sum_{k=0}^{L}\left(c_{ukx}^{\phi }C_{\tau -k}^{\phi }+p_{ukx}^{\phi }P_{\tau -k}^{\phi }\right)+e_{ux}$$

The parameters of the BFN influence the decoding results by altering the intercept and the slope in the Level 1 equation. Here, we take the regression equation of the X-axis as an example to explain the equation parameters in Level 2. Φ(Φ = {δ, θ, α^1^, α^2^, β^1^, β^2^, γ^1^, γ^2^})still represents the eight characteristic frequencies. *L* is the number of the intercepted segment EEG data in a time unit. τ−*k* represents the time period corresponding to *k* time units before the current time point τ (time point *t*−*s*). In Equation (12), $C_{\tau -k}^{\Phi }$ and $C_{\tau -k}^{\Phi }$ are the clustering coefficient and the average path length of the Φ band BFN corresponding to time point τ−*k*, respectively. $c_{vkx}^{\phi }$and $p_{vkx}^{\phi }$ are the slopes. *e*_*ux*_ is the residual. Equation (11) is the regression equation of the intercept. In Level 1, the intercept *V*_*x*_ does not have a direct operational relation with the characteristic frequencies, and hence the independent variable is the average of the network parameters corresponding to each frequency. Therefore, *C*_τ−*k*_ and*P*_τ−*k*_ are the mean values of $C_{\tau -k}^{\phi }$ and $P_{\tau -k}^{\phi }$ in the range Φ = {δ, θ, α^1^, α^2^, β^1^, β^2^, γ^1^, γ^2^}, respectively. *c*_*vkx*_ and *p*_*vkx*_ are the slopes, and *e*_*vx*_ is the residual.

## Results

### Calculation data

#### Network characteristic parameters

Experiment 1 comprised three motion modes: UD, LR, and BF. The purpose of Experiment 1 was to find the most sensitive EEG component for recognizing hand movement directions. First, the BFNs in unit time corresponding to eight EEG characteristic frequencies under the three motion modes were constructed (unit time was 2 s; EEG sampling frequency was 1,000 Hz). The network structure parameter vector *F*_*Net*_ is defined as follows:

(13) $${\text{F}}_{\text{Net}}=\{\text{DVector, VDegree, VPLength, Cluster}\}.$$

The nodes in the BFN represent the electrodes used in the EEG measurement. *DVector* represents the node degree vector formed by the 25 electrode nodes. *VDegree* represents the average of the node degrees. *VPLength* is the average path length, and *Cluster* is the clustering coefficient. The BFN structure parameter vector set was acquired using the motion mode as the data grouping condition, from which the node degree vectors were extracted and averaged to compute the node degree average vector for each motion mode.

**Figure 5:**
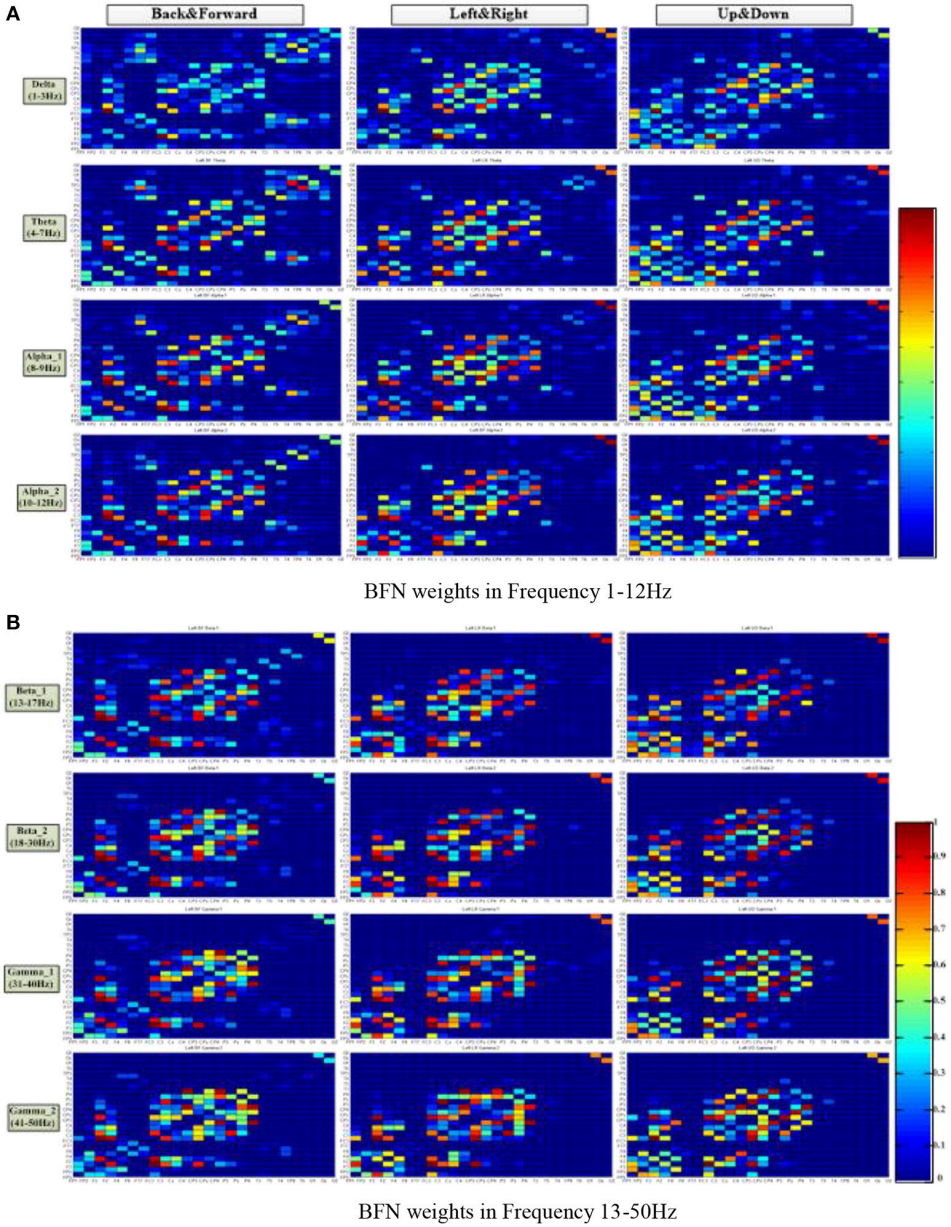
Distribution of the brain function network connection weight matrix in three motion patterns and eight frequencies. The columns represent the motion mode tags, and the rows represent the frequency tags. The color turned from red to blue in a gradient, representing a gradual decrease of connection weight, ranging from 1 to 0. **(A)** BFN weights at frequencies 1–12 Hz. **(B)** BFN weights at frequencies 13–50 Hz.

Figure 5 shows the average connection weight matrixes of the BFNs of the ten subjects in the three motion modes and eight frequencies. For each subgraph, the vertical coordinates from front to back are in the order of frontal, central, parietal, temporal, and occipital lobes. The horizontal coordinates from bottom to top are in the same order. In general, within the scope of the central lobe, the spectral correlation between each pair of EEG electrodes was strongest and was most relevant. The next is the frontal lobe. Among the brain areas, the central lobe and the frontal lobe are more relevant. From this figure, it can be seen that regardless of the motion mode, the number of connections in the BFNs decreased with an increase in the EEG frequencies. However, the variation between the connection weights increased gradually (the red color blocks, representing a value approaching 1, and the blue color blocks, representing a value approaching 0, increase simultaneously). The degree of difference was defined as the difference between the maximum connection weight and the nonzero minimum connection weight. The BFNs of different frequencies for the three motion modes showed significant differences in the variance of degrees (*p* < 0.05). The ones corresponding to frequencies delta and theta had greater degree of variance (*p* < 0.05), and the ones corresponding to beta_1 and beta_2 had smaller degrees of variance.

Table 2 shows the average node degree corresponding to each motion mode. The average node degree indicated the prevailing amount of correlation between the electrodes in the BFN. The largest standard deviation (SD) appeared in the BFN corresponding to frequency theta (*p* < 0.05), indicating that the changes in the motion modes had the maximum influence on the correlation between the electrodes in this network.

**Table 2:** Average degree corresponding to the three motion modes.

**Frequency**	**Node average degree**			***SD***
	**BF**	**LR**	**UD**	
Delta (1–3 Hz)	4.24	4.27	4.31	0.035
Theta (4–7 Hz)	4.41	4.27	4.46	*0.098*
Alpha_1 (8–9 Hz)	4.32	4.35	4.48	0.085
Alpha_2 (10–12 Hz)	4.31	4.37	4.45	0.070
Beta_1 (13–17 Hz)	4.30	4.29	4.39	0.055
Beta_2 (18–30 Hz)	4.32	4.26	4.30	0.031
Gamma_1 (31–40 Hz)	4.34	4.24	4.38	0.072
Gamma_2 (41–50 Hz)	4.29	4.33	4.43	0.072

*Underline denote biggest values in the respective column*.

Table 3 showed the average path length corresponding to each motion mode, representing the smallest cost for any two lead signals to have a correlation. The largest standard error appeared in the BFN corresponding to frequency alpha2 (*p* < 0.05), indicating that the changes in the motion modes had the maximum influence on the correlation between any two leads in this network.

**Table 3:** Average path length corresponding to the three motion modes.

**Frequency**	**Average path length**			***SD***
	**BF**	**LR**	**UD**	
Delta (1–3 Hz)	2.08	2.04	2.13	0.045
Theta (4–7 Hz)	2.18	2.09	2.37	0.143
Alpha_1 (8–9 Hz)	2.08	2.23	2.32	0.121
Alpha_2 (10–12 Hz)	2.02	2.15	2.35	*0.166*
Beta_1 (13–17 Hz)	1.89	2.07	2.16	0.137
Beta_2 (18–30 Hz)	1.85	1.94	2.07	0.111
Gamma_1 (31–40 Hz)	1.83	2.03	2.10	0.140
Gamma_2 (41–50 Hz)	1.88	1.95	2.14	0.135

*Underline denote biggest values in the respective column*.

Table 4 shows the clustering coefficient corresponding to each motion mode, indicating the level of the lead correlation aggregation. The largest standard error appeared in the BFN corresponding to frequency delta (*p* < 0.05), indicating that the changes in the motion modes had the maximum influence on the characteristic of the highly relevant lead clusters.

**Table 4:** Clustering coefficient corresponding to the three motion modes.

**Frequency**	**Clustering coefficient**			***SD***
	**BF**	**LR**	**UD**	
Delta (1–3Hz)	0.49	0.51	0.51	0.012
Theta (4–7 Hz)	0.51	0.51	0.52	0.006
Alpha_1 (8–9 Hz)	0.51	0.50	0.51	0.006
Alpha_2 (10–12 Hz)	0.50	0.49	0.49	0.006
Beta_1 (13–17 Hz)	0.49	0.50	0.49	0.006
Beta_2 (18–30 Hz)	0.51	0.49	0.5	0.010
Gamma_1 (31–40 Hz)	0.51	0.49	0.5	0.010
Gamma_2 (41–50 Hz)	0.51	0.50	0.5	0.006

#### Key electrode node

In the three motion modes, the standard error of the *DVector* averaged on all unit times was computed, and the values are shown in Table 5. Standard error measures how far from the mean a set of numbers is distributed. A large standard error indicates that most of the values are far from the mean; a small standard error indicates that the numbers are close to the mean. Standard error was used here to represent the influence of different motion modes on the node degree in the BFN at a given frequency and also manifested the characterization ability of the BFN in a motion mode.

**Table 5:** Mean values of standard deviations of node degrees.

	**Delta**	**Theta**	**Alpha1**	**Alpha2**	**Beta1**	**Beta2**	**Gamma1**	**Gamma2**
Fp1	0.68	0.75	0.73	0.72	0.76	0.74	0.74	0.90
FP2	0.86	1.16	1.13	1.03	1.07	0.68	0.57	0.62
F3 FZ	0.10 0.43	0.29 0.50	0.28 0.24	0.31 0.52	0.20 0.27	0.28 0.32	0.28 0.54	0.13 0.38
F4	0.71	0.70	0.96	0.95	1.12	0.94	0.36	0.40
F8	0.88	1.09	0.67	0.47	0.38	0.34	0.40	0.37
FT7	0.63	0.46	0.44	0.45	0.30	0.20	0.29	0.22
FC3	0.16	0.32	0.21	0.26	0.27	0.40	0.17	0.20
C3 Cz	0.89 0.91	0.63 0.89	0.62 0.81	0.37 0.85	0.27 0.67	0.59 0.68	0.55 0.61	0.66 0.64
C4 CP3	0.75 0.71	0.71 0.56	0.52 0.36	0.32 0.17	0.50 0.20	0.26 0.28	0.15 0.63	0.20 0.81
CPz	0.72	0.60	0.41	0.39	0.16	0.49	0.98	1.14
CP4	0.58	0.51	0.03	0.22	0.50	0.71	0.62	0.82
P3	0.38	0.41	0.19	0.13	0.32	0.57	0.56	0.11
Pz	0.53	0.16	0.17	0.10	0.31	0.18	0.28	0.55
P4	0.21	0.26	0.27	0.24	0.40	0.28	0.31	0.52
T3	0.95	0.83	0.52	0.60	0.43	0.24	0.14	0.09
T5	1.24	0.88	0.53	0.71	0.47	0.13	0.06	0.07
T4	1.4	1.6	1.22	0.72	0.34	0.26	0.22	0.28
TP8	0.78	1	0.63	0.64	0.23	0.08	0.060	0
T6	0.53	0.36	0.07	0.15	0.03	0.02	0.02	0.04
O1	0.75	0.44	0.32	0.41	0.09	0.21	0.22	0.18
Oz	0.61	0.53	0.60	0.61	0.45	0.50	0.37	0.32
O2	0.05	0.12	0.53	0.42	0.26	0.32	0.19	0.13
Mean	0.66	0.63	0.50	0.47	0.40	0.39	0.37	0.39

In Table 5, the rows represent the 25 electrode nodes, and the columns represent the eight EEG characteristic frequencies. The “mean” in the last row is the average standard error of the average node degree mean of the BFN for the characteristic frequencies. Delta (1–3 Hz) obtained the highest average standard error of 0.66, followed by theta (4–7 Hz) with a standard error of 0.63, alpha1 (8–9 Hz) with 0.50, alpha2 (10–12 Hz) with 0.47, beta1 (13–17 Hz) with 0.4, beta2 (18–30 Hz) with 0.39, gamma2 (41–50 Hz) with 0.39, and gamma1 (31–40 Hz) with 0.37. The average standard error showed a decreasing trend from low to high frequencies. Thus, we concluded that the node degree variation in a low-frequency BFN is more sensitive to the changes in the motion modes.

#### Key electrode frequency

The node degree vectors were grouped by the motion mode and tested for significance. The Kruskal–Wallis test was used to compute the *P*-value to determine the characteristic EEG frequencies and the electrodes that were sensitive to the hand motion direction. The Kruskal–Wallis test is a nonparametric test developed on the basis of the two-independent-sample Mann–Whitney *U*-test for multiple samples. It could also be used to test whether the distribution of multiple samples had a significant variance. First, the data of all the samples were combined and arranged in the ascending order. The variables were then ranked, and the average rank of each group was tested for significant variance. If there was no significant difference between the mean ranks of the groups, it was because the data from the groups were well mixed and there was no significant difference in the data values; thus, it could be concluded that the distributions of the populations had no significant variance. In contrast, if there were significant differences among the mean ranks of the groups, it was because the data from different groups could not be well mixed—some groups had larger data values, while others had smaller data values. Thus, it could be concluded that the distribution of the different populations had a significant variance, *p* < 0.05.

The significance analysis results in Table 6 show that for frequency delta, 14 electrodes showed significant differences in the node degree; for frequency theta, 15 electrodes showed significant differences; for frequency gamma1, 14 electrodes showed significant differences. The other BFNs had too few nodes with significant differences (less than half of the total leads); therefore, the EEG components and the corresponding network parameters of these frequencies were not included in the following hand trajectory decoding model training. The values of 0 and 1 in the table were used because the node degree was always 0, which was different from *p* < 0.05. Electrodes with *p* < 0.05 for all the three frequencies delta, theta, and gamma1 were FP1, F4, F8, C3, Cz, C4, CP4, T3, and T4. The EEG signals from FP1 were affected by the elctrooculogram. So the EEG signals from FP1 were excluded from the decoding model training. The characteristic electrodes selected for training the hand trajectory decoding model were F4, F8, C3, Cz, C4, CP4, T3, and T4.

**Table 6:** *P*-values from Kruskal–Wallis test of node degree vectors.

	**Delta**	**Theta**	**Alpha_1**	**Alpha_2**	**Beta_1**	**Beta_2**	**Gamma_1**	**Gamma_2**
FP1	0.03	0.007	0.006	0.013	0.003	0.003	0.006	0.001
Fp2	0.001	0	0	0	0	0.002	0.006	0.003
F3	0.856	0.029	0.379	0.254	0.76	0.531	0.376	0.769
Fz	0.218	0.099	0.738	0.195	0.809	0.316	0.025	0.285
F4	0.032	0.003	0	0	0	0	0.032	0.013
F8	0.001	0	0	0.011	0.024	0.035	0.009	0.008
FT7	0.002	0.09	0.267	0.112	0.141	0.001	0	0.002
FC3	0.843	0.149	0.633	0.33	0.282	0.168	0.661	0.34
C3	0.009	0.024	0.025	0.213	0.594	0.006	0.023	0.072
Cz	0.006	0.014	0.051	0.006	0.055	0.05	0.033	0.016
C4	0.029	0.008	0.018	0.307	0.016	0.147	0.048	0.756
CP3	0.057	0.017	0.269	0.714	0.613	0.158	0.001	0
CPz	0.054	0.005	0.215	0.529	0.918	0.079	0.002	0
CP4	0.003	0.009	0.893	0.83	0.422	0.014	0.007	0.006
P3	0.17	0.034	0.23	0.656	0.606	0.319	0.5	0.568
Pz	0.058	0.739	0.674	0.675	0.437	0.801	0.568	0.053
P4	0.714	0.326	0.268	0.237	0.009	0.013	0.071	0
T3	0.001	0.015	0.008	0	0	0.005	0.016	0.152
T5	0	0	0.009	0.004	0.003	0.102	0.604	0.23
T4	0.007	0.024	0	0	0	0.005	0.005	0.005
TP8	0	0	0	0	0.001	0.064	0.165	1
T6	0.003	0.033	0.217	0.238	0.348	0.368 0	0.368	0.132
O1	0.023	0.008	0.002	0.001	0.1891	0	0
Oz	0.005	0.003	0	0	0	0	0	0
O2	0.834	0.805	0	0	0.009	0	0.067	0.248

*Underline denote biggest values in the respective column*.

### Free trajectory decoding

#### Method of evaluation

The decoding model of voluntary hand movement is a hierarchical linear regression model. In the first level, the coordinates of hand motion on the X-, Y-, and Z-axes were dependent variables, and the characteristic EEG components were independent variables. In the second level, the BFN parameters (average path length and clustering coefficient) corresponding to the characteristic frequencies were independent variables, while the intercept and the slope of the regression equation were dependent variables. As mentioned previously, the most sensitive EEG components to recognize the hand motion direction were distributed for frequencies delta, theta, and gamma1. The characteristic electrodes screened out were F4, F8, C3, Cz, C4, CP4, T3, and T4.

In the experiment, only the motion data of the right hand were recorded. Five of the ten subjects, who provided more sensitive EEG components for recognizing the hand motion direction, participated in the spiral trajectory decoding test. Each subject generated 40 groups of EEG-trajectory synchronous data for each spiral trajectory. For each subject, the average accuracy was over 10 runs of the 10-fold cross-validation procedure. Then, we combined five subjects' EEG-trajectory synchronous data into a large dataset, and carried out 10 runs of the 10-fold cross validation procedure to verify the decoding accuracy of the proposed method. During the test, continuous decoding calculation based on multiple linear regression (Bradberry et al., 2009, 2010) was used, and the results were compared with the decoding results of this two-level linear regression model. The Pearson correlation coefficient (PCC) was introduced in the evaluation. The testing results were evaluated by computing the correlation coefficient between the decoding trajectory and the measurement trajectory. The equation for calculating the Pearson correlation coefficient *R* is

(14) $$\begin{array}{l} \;R=avg.\left(R_{x},R_{y},R_{z}\right),\\ R_{i}\left(I,\hat{I}\right)=\frac{cov\left(I,\hat{I}\right)}{\sigma_{I}\sigma_{\hat{I}}},\;i\in \left\{x,y,z\right\}. \end{array}$$

In the equation, *R* is the average value of the Pearson correlation coefficient on the three axes (X, Y, and Z). Î is the coordinates of the decoded trajectory on the corresponding axis; *I* is the coordinates of the measurement trajectory on the corresponding axis. σ_*I*_and σ_Î_ are the standard errors of *I* and Î. Before calculating the correlation coefficient, the decoding trajectory was processed through a fourth-order low-pass Butterworth filter, smoothing the signal at a cutoff frequency of 1 Hz. The mean of the correlation coefficient of each subject was calculated to evaluate the test results.

#### Spiral trajectory decoding

Table 7 shows the result of the multiple linear regression method in the first spiral trajectory test, and the average correlation coefficient between the decoded trajectories and the measured paths from each subject. Subject 1 obtained the largest average correlation coefficient of 0.6397 on the three axes. The average correlation coefficient of all the subjects on the three axes was 0.5125.

**Table 7:** The average PCC of spiral trajectory I from multiple linear regression model.

**PCC**	**Subject1**	**Subject2**	**Subject3**	**Subject4**	**Subject5**	**Mean**
*R*_*x*_	0.7348	0.3481	0.4722	0.4199	0.4647	0.4824
*R*_*y*_	0.5781	0.4872	0.6118	0.4772	0.6108	0.5465
*R*_*z*_	0.6061	0.4369	0.3728	0.5788	0.4886	0.4257
*R*	0.6397	0.4241	0.4856	0.4920	0.5213	0.5125

*Underline denote biggest values in the respective column*.

Table 8 shows the result of the hierarchical linear regression method in the first spiral trajectory test, and the average correlation coefficient between the decoded trajectory and the measured path of the 10 × 10-fold cross-validation procedure for each subject. Subject 4 obtained the highest average correlation coefficient of 0.8606 on the three axes. The average correlation coefficient of all subjects on the three axes was 0.6939, which was better than the result of the multiple linear regression model.

**Table 8:** The average PCC of spiral trajectory I from hierarchical linear regression model.

**PCC**	**Subject1**	**Subject2**	**Subject3**	**Subject4**	**Subject5**	**Mean**
*R*_*x*_	0.5908	0.5895	0.5186	0.8233	0.7821	0.6609
*R*_*y*_	0.8625	0.8224	0.8244	0.9955	0.7568	0.8523
*R*_*z*_	0.6171	0.6735	0.5041	0.8712	0.6392	0.6610
*R*	0.6710	0.6332	0.5927	0.8606	0.7118	0.6939

*Underline denote biggest values in the respective column*.

Tables 9, 10 show the results of multiple linear regression and hierarchical linear regression, respectively, in the second spiral trajectory testing. Subject 3 obtained the highest average correlation coefficients in both methods: 0.8721 and 0.6213, respectively. The multiple linear regression generated an average correlation coefficient of 0.5264 for all subjects on the three axes. The hierarchical linear regression obtained was 0.6871, which still showed an advantage in terms of decoding efficiency. Then, we combined the EEG-trajectory synchronous data of five subjects into a large dataset, and carried out 10 runs of the 10-fold cross validation procedure to verify the decoding accuracy of the proposed method. The results of multiple linear regression and hierarchical linear regression were 0.5721 and 0.7315, respectively. The hierarchical linear regression still obtained a better result. To evaluate the effects of the hierarchical linear regression model using the ROC curve, the reference value range of the original data and regression forecast values were 95%. If the observations for the participants were within the reference value range, they would be determined to be normal and recorded as “zero;” otherwise, they would be determined to be abnormal and as “one.” Then the estimations of the both original observations and forecast values were performed using the ROC curve. At the end, we obtained AUC = 0.819.

**Table 9:** The average PCC of spiral trajectory II from multiple linear regression model.

**PCC**	**Subject1**	**Subject2**	**Subject3**	**Subject4**	**Subject5**	**Mean**
*R*_*x*_	0.8826	0.4059	0.7592	0.5715	0.6792	0.6597
*R*_*y*_	0.3004	0.4440	0.4844	0.3205	0.2503	0.3600
*R*_*z*_	0.5058	0.5296	0.6203	0.6598	0.4822	0.5595
*R*	0.5629	0.4598	0.6213	0.5172	0.4706	0.5264

*Underline denote biggest values in the respective column*.

**Table 10:** The average PCC of spiral trajectory II from hierarchical linear regression model.

**PCC**	**Subject1**	**Subject2**	**Subject3**	**Subject4**	**Subject5**	**Mean**
*R*_*x*_	0.5213	0.6084	0.9062	0.4426	0.7966	0.6550
*R*_*y*_	0.7017	0.6085	0.7979	0.3297	0.4740	0.5824
*R*_*z*_	0.6887	0.8823	0.9123	0.8826	0.7545	0.8241
*R*	0.6372	0.6997	0.8721	0.5516	0.6750	0.6871

*Underline denote biggest values in the respective column*.

**Figure 6:**
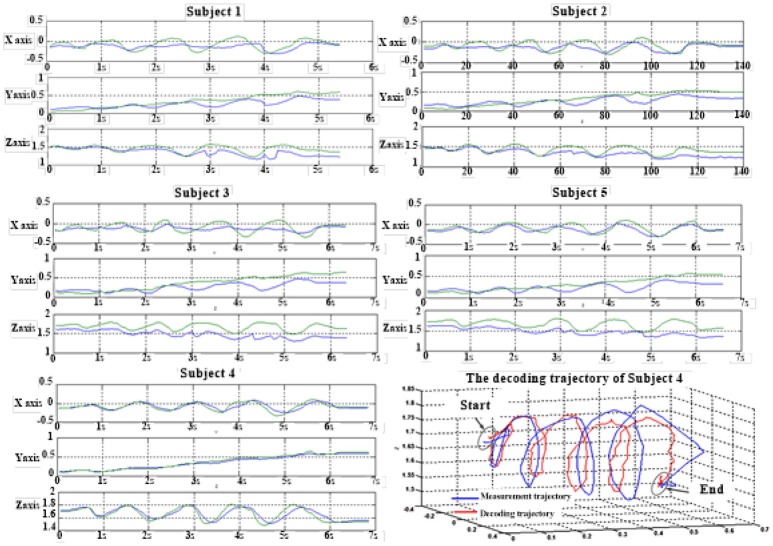
Test results of spiral trajectory I of five subjects.

**Figure 7:**
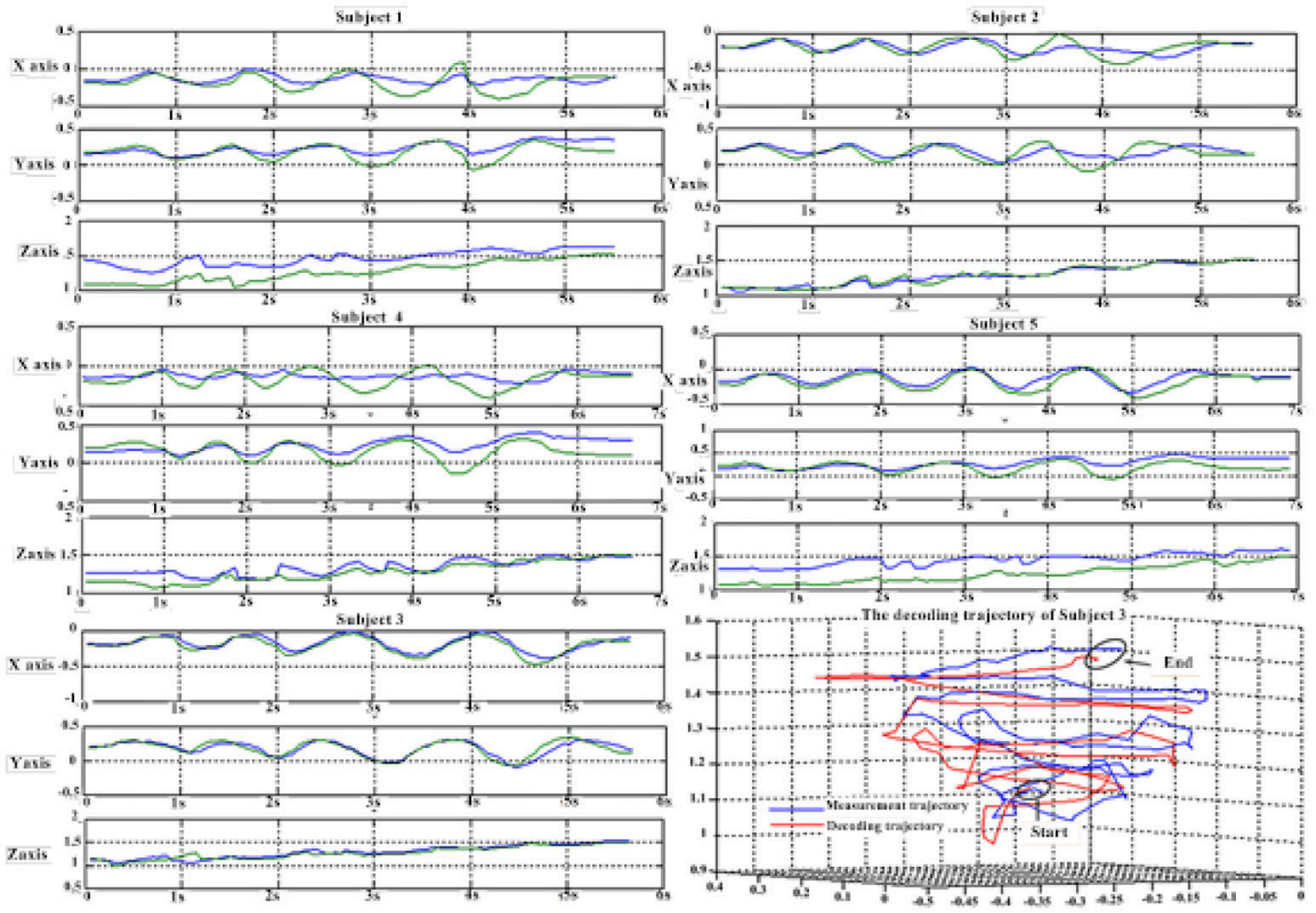
Test results of the spiral trajectory II.

Figure 6 depicts the best decoding trajectories of each subject for the first spiral trajectory. Subject 4 obtained the best testing results, of which the complete decoding trajectory is illustrated in the lower right corner of Figure 6. Figure 7 shows the test result of the second spiral trajectory. Subject 3 obtained the best testing results, of which the complete decoding path is illustrated in the lower right corner.

## Discussion

### Experimental paradigm

In this study, we focused on voluntary hand movement decoding. A 40-channel NuAmps system (NeuroScan, Inc. Sterling, USA) was used for data recording at a sampling rate of 1,000 Hz. We used the PST IRIS optical tracking/measurement system (PS-tech, Amsterdam, Netherlands) to measure and record the kinematics parameters of voluntary hand movement. The sampling rate was 120 Hz. The BFNs in unit time corresponding to eight EEG characteristic frequencies under three motion modes were constructed. The unit time was 2 s. As the result of feature extraction, the characteristic frequencies were delta, theta, and gamma1. The characteristic electrodes selected for training the hand trajectory decoding model were F4, F8, C3, Cz, C4, CP4, T3, and T4.

In the research field of neural prosthesis, it is critical that robots follow real body movements. Because BCIs do not depend on neuromuscular control, BCIs provide options for communication and control for people with devastating neuromuscular disorders (such as amyotrophic lateral sclerosis, or ALS, brainstem stroke, cerebral palsy, and spinal cord injury). Voluntary movement is the expression of thought through action, in which virtually all areas of the central nervous system are involved. Compared with certain restricted motion modes, e.g., center-out reaching task and limb movement according to cue, the motion of the human hand is in a “half-goal-directed” mode. In this study, subjects swung their right hands according to their will. The original purpose of the experiment was to reduce the cognitive load in motion. The components related to limb motion in EEG were anticipated to be relatively stronger than the components extracted in the paradigm of nonvoluntary movement.

Compared to previous EEG-based limb motion decoding studies, this study used a smaller sample size and a smaller number of electrodes because of the experimental paradigm and the feature extraction method used in this study. Prior to the formal experiment, all subjects participated in the preliminary training of hand movement. The most important constraint of experiment 1 was that subjects moved their hands in one axis while preventing the movement in the other two axes. After data collection, we calculated the variance of the coordinate values of the measurement trajectory in the X-, Y-, and Z axes respectively. If the variance was substandard, the corresponding EEG-trajectory data were not included in subsequent calculations. Therefore, the initial purpose of this study was to extract the most sensitive EEG components and the minimum EEG feature sets for recognizing hand movement directions. This is conducive to the current approach to application transformation. However, from another perspective, if low-resolution EEG is used, there would be possible defects in practical applications. The EEG components strongly related to changes in emotions may miss. Second, when the motion command is output, there will be a situation in which the motion control of external device (e.g., a humanoid robot) is not continuous. For this scenario, we envision a kinematic approach to realize smooth motion control of the robotic arm.

### Brain connectivity analysis using EEG

On the basis of wavelet analysis, the correlation coefficients of the wavelet coefficients from 25 electrodes were calculated for eight characteristic frequencies. Combining the basic theory of the BFN, we attempted to explore the cooperative relationship among the different brain regions in the process of voluntary movement. Four key parameters of the BFN were investigated, namely node degree, average of node degree, average path length, and clustering coefficient. By analyzing the node degree, we found that the BFNs using low-frequency EEG signals contained more network connections. For the three motion modes, the degree of variance among the connection weights for the frequencies delta (1–3 Hz) and theta (4–7 Hz) were greater. This result revealed that the node degree variation in a low-frequency BFN was more sensitive to the changes in the motion modes. This is in line with the conclusion of Contreras-Vidal that low-frequency EEG and EcoG signals (<3 Hz) had special significance in finger motion trajectory decoding. Through a Kruskal–Wallis test of the node degree vectors, we found that 14 electrodes in the delta BFN exhibited significant differences in the node degree; 15 electrodes in the theta BFN showed significant differences, and 14 electrodes in the gamma1 BFN exhibited significant differences. This result provided evidence that the high frequency also played a central role in voluntary hand movement decoding. This conclusion agrees well with the research result of Korik (Korik et al., 2014) that the frequency range of 28–36 Hz can be used to decode voluntary hand movements, besides the frequency 0.4–4 Hz. Finally, the most sensitive components used in decoding were the EEG signals from electrodes F4, F8, C3, Cz, C4, CP4, T3, and T4 for frequencies delta, theta, and gamma1. The electrodes were selected according to the *P*-values obtained from the Kruskal–Wallis test. Among these electrodes, C3 and C4, which are located on the primary somatosensory cortex, are responsible for hand movement, consistent with the result of Izabela's study (Izabela, 2013). The location of electrode CP4, corresponding approximately to the motor cortex and the somatosensory cortex, is determined by the decoding model as critical for hand movement (Christine et al., 2015). Electrodes T3 and T4 are related to the verbal analytical process and visual-spatial processing. Electrodes F4 and F8 are related to the selection of movements and the voluntary control of body movement, respectively (Machado et al., 2013).

### Motion decoding model

Some underlying shortcomings of EEG present several challenges to voluntary movement decoding research based on EEG. To overcome these challenges and obtain better decoding results, we applied a method to expand the types of features extracted from EEG. In this research, we proposed a model of voluntary hand movement decoding based on an HLM. The original intention of this design was to identify a computing architecture that could contain and describe complex data, such as that of an EEG BFN. Hierarchical linear modeling is an ordinary least squares regression-based analysis that takes the hierarchical structure of the data into account. Hierarchically structured data are nested data where groups of units are clustered together in an organized fashion, such as students within classrooms within schools. In this work, we adjusted the EEG data and the BFN parameters into a nested structure based on actual data relationships, such as BFN parameters within some certain frequency bands of the EEG signals obtained from electrodes. Based on hierarchical linear regression, we designed a voluntary movement decoding model using EEG data and BFN parameters as inputs. In the first level of the decoding model, the coordinates of the hand were the dependent variables. EEG data were independent variables. In the second level, the parameters of the BFN influence the decoding results by altering the intercept and the slope in the Level 1 equation. We used the clustering coefficient and the average path length as independent variables. As the test results demonstrated, the decoding model based on the HLM obtained superior results compared to the multiple linear regression model. In future work, a method of deep learning will be introduced into data processing, e.g., CNN. It is expected that this approach will open new vistas for decoding voluntary limb movements.

## Author contributions

TL was mainly responsible for designing experiments, data analysis and writing paper. TX was mainly responsible for organizing the experimental environment and equipment. BW was mainly responsible for experimental implementation. JZ was mainly responsible for general guidance and project management.

### Conflict of interest statement

The authors declare that the research was conducted in the absence of any commercial or financial relationships that could be construed as a potential conflict of interest.

## References

[B1] Babiloni C. Percio C. D. Lopez S. Gennaro G. D. Quarato P. P. Pavone L.. (2017). Frontal functional connectivity of electrocorticographic delta and theta rhythms during action execution versus action observation in humans. Front. Behav. Neurosci. 11:20. 10.3389/fnbeh.2017.00020 28223926 PMC5294389

[B2] Boostani R. Moradi M. H. (2003). Evaluation of the forearm EMG signal features for the control of a prosthetic hand. Physiol. Meas. 24, 309–319. 10.1088/0967-3334/24/2/307 12812417

[B3] Bradberry T. J. Gentili R. J. Contreras-Vidal J. L. (2010). Reconstructing three-dimensional hand movements from noninvasive electroencephalographic signals. J. Neurosci. 30, 3432–3437. 10.1523/JNEUROSCI.6107-09.2010 20203202 PMC6634107

[B4] Bradberry T. J. Rong F. Contreras-Vidal J. L. (2009). Decoding center-out hand velocity from MEG signals during visuomotor adaptation. NeuroImage 47, 1691–1700. 10.1016/j.neuroimage.2009.06.023 19539036

[B5] Breitwieser C. Kreilinger A. Neuper C. Müller-Putz G. R. (2010). The TOBI hybrid BCI - the data acquisition module in Proceedings of the First TOBI Workshop (Graz).

[B6] Cavallo A. Koul A. Ansuini C. Capozzi F. Becchio C. (2016).Decoding intentions from movement kinematics. Sci. Rep. 6:37036 10.1038/srep37036 27845434 PMC5109236

[B7] Chaudhary U. Xia B. Silvoni S. Cohen L. G. Birbaumer N. (2017). Brain–computer interface–based communication in the completely locked-in state. PLoS Biol. 15:e1002593. 10.1371/journal.pbio.1002593 28141803 PMC5283652

[B8] Christine E. K. Po T. W. Colin M. M. Cathy C. Y. Chou A. H. D. Zoran N. (2015). The feasibility of a brain-computer interface functional electrical stimulation system for the restoration of over ground walking after paraplegia. J. Neuro Eng. Rehabil. 12:80 10.1186/s12984-015-0068-7 PMC4581411 26400061

[B9] Coyle S. M. Ward T. E. Markham C. M. (2007). Brain-computer interface using a simplified functional near-infrared spectroscopy system. J. Neural Eng. 4, 219–226. 10.1088/1741-2560/4/3/007 17873424

[B10] Decety J. (1995). The neurophysiological basis of motor imagery. Behav Brain Res. 77, 45–52. 10.1016/0166-4328(95)00225-1 8762158

[B11] Filho C. A. S. Attux R. Castellano G (2018). Can graph metrics be used for EEG-BCIs based on hand motor imagery? Biomed. Signal Process. Control 40, 359–365. 10.1016/j.bspc.2017.09.026

[B12] He'tu S. Gregoire M. Saimpont A. Coll M. P. Eugène F. Michon P. (2013). The neural network of motor imagery: an ALE meta-analysis. Neurosci. Biobehav. Rev. 37, 930–949. 10.1016/j.neubiorev.2013.03.017 23583615

[B13] Hochberg L. R. Bacher D. Jarosiewicz B. Masse N. Y. Simeral J. D. Vogel J.. (2012). Reach and grasp by people with tetraplegia using a neurally controlled robotic arm. Nature 485, 372–375. 10.1038/nature11076 22596161 PMC3640850

[B14] Ibáñez J. Serrano J. I. Del Castillo M. D. Monge-Pereira E. Molina-Rueda F. Alguacil-Diego I.. (2014). Detection of the onset of upper-limb movements based on the combined analysis of changes in the sensorimotor rhythms and slow cortical potentials. J. Neural Eng. 11:056009. 10.1088/1741-2560/11/5/056009 25082789

[B15] Izabela R. (2013). Genetic algorithms in EEG feature selection for the classification of movements of the left and right hand in Proceedings of the 8th International Conference on Computer Recognition Systems CORES (Milkow), 579–589.

[B16] Jochumsen M. Niazi I. K. Dremstrup K. Kamavuako E. N. (2016). Detecting and classifying three different hand movement types through electroencephalography recordings for neurorehabilitation. Med. Biol. Eng. Comput. 54, 1491–1501. 10.1007/s11517-015-1421-5 26639017

[B17] Khan M. J. Hong M. J. Hong K. S. (2014). Decoding of four movement directions using hybrid NIRS-EEG brain-computer interface. Front. Hum. Neurosci. 8:244. 10.3389/fnhum.2014.00244 24808844 PMC4009438

[B18] Korik A. Siddique N. H. Sosnik R. Coyle D. (2014). Correlation of EEG band power and hand motion trajectory in 6th International BCI Conference 2014. Graz University of Technology (Graz), 1–4.

[B19] Lacourse M. G. Turner J. A. Randolph-Orr E. Schandler S. L. Cohen M. J. (2004). Cerebral and cerebellar sensorimotor plasticity following motor imagery-based mental practice of a sequential movement. J. Rehabil. Res. Dev. 41, 505–524. 10.1682/JRRD.2004.04.0505 15558380

[B20] Lal T. N. Hinterberger T. Widman G. Schroeder M. Hill J. (2005). Methods towards invasive human brain computer interfaces. Adv. Neural Inform. Process. Syst. 17, 737–744.

[B21] Lew E. Chavarriaga R. Silvoni S. Del R. Millán J. (2012). Detection of self-paced reaching movement intention from EEG signals. Front. Neuroeng. 5:13. 10.3389/fneng.2012.00013 23055968 PMC3458432

[B22] Liao K. Xiao R. Gonzalez J. Ding L. (2014).Decoding individual finger movements from one hand using human EEG signals. PLoS ONE 9:e85192 10.1371/journal.pone.0085192 24416360 PMC3885680

[B23] Machado D. C. D. Lima G. C. dos Santos R. S. Ramos A. J. B. de Sousa C. C. M. dos Santos R. P. M. (2013). Electroencephalographic analysis in left hemiparesis: a case study. Rev. Bras. Neurol. 49, 129–136.

[B24] Miller K. J. Schalk G. Fetz E. E. den Nijs M. Ojemann J. G. Rao R. P.. (2010). Cortical activity during motor execution, motor imagery, and imagery-based online feedback. Proc. Natl. Acad. Sci. U.S.A. 107, 4430–4435. 10.1073/pnas.0913697107 20160084 PMC2840149

[B25] Min B. K. Marzelli M. J. Yoo S. -S. (2010). Neuroimaging-based approaches in brain-computer interface. Trends Biotechnol. 28, 552–560. 10.1016/j.tibtech.2010.08.002 20810180

[B26] Müller-Putz G. R. Schwarz A. Pereira J. Ofner P. (2016). From classic motor imagery to complex movement intention decoding: the noninvasive Graz-BCI approach. Prog. Brain Res. 228, 39–70. 10.1016/bs.pbr.2016.04.017 27590965

[B27] Nair D. G. Purcott K. L. Fuchs A. Steinberg F. Kelso J. A. (2003). Cortical and cerebellar activity of the human brain during imagined and executed unimanual and bimanual action sequences: a functional MRI study. Cogn. Brain Res. 15, 250–260. 10.1016/S0926-6410(02)00197-0 12527099

[B28] Nicolas-Alonso L. F. Gomez-Gil J. (2012). Brain-computer interfaces, a review. Sensors 12, 1211–1279. 10.3390/s120201211 22438708 PMC3304110

[B29] Pereira J. Ofner P. Schwarz A. Sburlea A. I. Müllerputz G. R. (2017). EEG neural correlates of goal-directed movement intention. Neuroimage 149, 129–140. 10.1016/j.neuroimage.2017.01.030 28131888 PMC5387183

[B30] Pistohl T. Schulze-Bonhage A. Aertsen A. Mehring C. Ball T. (2012). Decoding natural grasp types from human ECoG. NeuroImage 59, 248–260. 10.1016/j.neuroimage.2011.06.084 21763434

[B31] Schalk G. Kubanek J. Miller K. J. Anderson N. R. Leuthardt E. C. Ojemann J. G. (2007).Decoding two-dimensional movement trajectories using electrocorticographic signals in humans. J. Neural Eng. 4, 264–275. 10.1088/1741-2560/4/3/012 17873429

[B32] Sitaram R. Caria A. Veit R. Gaber T. Rota G. Kuebler A. (2007). fMRI brain-computer interface: a tool for neuroscientific research and treatment. Comput. Intell. Neurosci. 2007, 1–10. 10.1155/2007/25487 PMC2233807 18274615

[B33] Sporns O. Tononi G. Kotter R. (2005). The human connectome: a structural description of the human brain. PLoS Comput. Biol. 1:e42. 10.1371/journal.pcbi.0010042 16201007 PMC1239902

[B34] Úbeda A. Azorín J. M. Chavarriaga R. Millán J. D. R. (2017). Classification of upper limb center-out reaching tasks by means of EEG-based continuous decoding techniques. J. Neuroeng. Rehabilit. 14:9. 10.1186/s12984-017-0219-0 28143603 PMC5286813

[B35] Wilson J. A. Felton E. A. Garell P. C. Schalk G. Williams J. C. (2006). ECoG factors underlying multimodal control of a brain-computer interface. IEEE Trans. Neural Syst. Rehabil. Eng. 14, 246–250. 10.1109/TNSRE.2006.875570 16792305

[B36] Wolpaw J. R. McFarland D. J. (2004). Control of a two-dimensional movement signal by a noninvasive brain-computer interface in humans. Proc. Natl. Acad. Sci. U.S.A. 101, 17849–17854. 10.1073/pnas.0403504101 15585584 PMC535103

[B37] Yoo S. S. Fairneny T. Chen N. K. Choo S. E. Panych L. P. Park H.. (2004). Brain-computer interface using fMRI: spatial navigation by thoughts. Neuroreport 15, 1591–1595. 10.1097/01.wnr.0000133296.39160.fe 15232289

[B38] Yu H. Liu J. Cai L. Wang J. Cao Y Hao C. (2017). Functional brain networks in healthy subjects under acupuncture stimulation: an EEG study based on nonlinear synchronization likelihood analysis. Physica A 468, 566–577. 10.1016/j.physa.2016.10.068

